# Multicenter phase II study of apatinib treatment for metastatic gastric cancer after failure of second-line chemotherapy

**DOI:** 10.18632/oncotarget.21053

**Published:** 2017-09-19

**Authors:** Hanguang Ruan, Junlin Dong, Xueliang Zhou, Juan Xiong, Hua Wang, Xiaoming Zhong, Xiaolong Cao

**Affiliations:** ^1^ Department of Oncology, The Third Hospital of Nanchang City, Jiangxi Province, Nanchang, China; ^2^ Graduate School of Jiangxi Medical College, Nanchang University, Nanchang, China; ^3^ Department of Oncology, Zhuhai Peoples’ Hospital of Guang Dong Province, Zhu Hai, China; ^4^ Cancer Centre, The First Affiliated Hospital of Nanchang University, Nanchang, China; ^5^ Department of Cancer Radiotherapy, Jiangxi Cancer Hospital of Jiangxi Province, Nanchang, China; ^6^ Department of Oncology, Panyu District Center Hospital of Guang Dong Province, Guangzhou, China

**Keywords:** Apatinib, metastatic gastric cancer (mGC), effective, adverse events

## Abstract

Apatinib is a tyrosine kinase inhibitor and vascular endothelial growth factor receptor 2 (VEGFR-2) targeted drug. A phase I clinical trial showed that this agent has antitumor activity in Chinese patients with metastatic gastric cancer (mGC). The aim of this study was to investigate the safety and efficacy of apatinib treatment in patients with mGC.

This was an open-label, multicenter, single-arm study involving four institutions in China. We enrolled 42 patients from March 2015 to October 2015 who experienced tumor progression after second-line chemotherapy and had no other treatment options that clearly conferred a survival benefit. Oral apatinib (850 mg daily) was administered within 30 min of eating breakfast, lunch, or dinner on days 1 through 28 of each 4-week cycle.

The median progression-free survival (PFS) time and median overall survival (OS) time were 4.0 months (95% CI, 2.85-5.15) and 4.50 months (95% CI, 4.03-4.97), respectively. The disease control rate (DCR) and objective response rate (ORR) were, respectively, 78.57% and 9.52% after 2 cycles and 57.14% and 19.05% after 4 cycles. The main adverse events (AEs) were secondary hypertension, elevated aminotransferase, and hand-foot syndrome, with incidences of 35.71%, 45.24%, and 40.48%, respectively. The most common grade 3 to 4 AEs were secondary hypertension and elevated aminotransferase, with incidences of 7.14% each.

Apatinib is effective and safe in heavily pretreated patients with mGC who fail to respond to two or more prior chemotherapy regimens. Toxicities were tolerable or could be clinically managed.

## INTRODUCTION

Gastric cancer is a common malignant tumor that is associated with a very high incidence and mortality rate in our country. Its rates of morbidity and mortality are higher in rural areas than in the city. Locally advanced or metastatic gastric cancer has been diagnosed in the vast majority of patients by the time they enter the hospital [1-3]. In these cases, a multidisciplinary team (MDT) is assigned and general treatment measures may become the main disease management approach. Some oncologists regard front-line chemotherapy as the standard therapeutic regimen for extending progression free survival and overall survival times in patients with metastatic gastric cancer (mGC). New evidence also suggests that second-line salvage chemotherapies confer a survival advantage when compared with the best supportive care. But for patients who fail second-line chemotherapy, further effective treatment is needed [4-12]. Molecular targeted therapy may become an effective treatment option for those patients [13-16].

Bevacizumab is a vascular endothelial growth factor (VEGF) inhibitor and widely used in the treatment of tumors, especially lung, breast, kidney, and liver. Its effectiveness in the treatment of colon cancer and other solid tumors has been demonstrated [17, 18]. Trastuzumab is an effective treatment in patients with Her-2 positive breast and gastric cancers [19-21]. Apatinib is a small-molecule VEGF receptor (VEGFR) tyrosine kinase inhibitor, similar to vatalanib (PTK787), and has 10 times the binding affinity of vatalanib or sorafenib. The drug has received intellectual property protection in our country as a new kind of oral molecular targeted drug. Investigators in China have carried out a phase I clinical trial and shown that this agent has antitumor activity in Chinese patients with mGC who failed second-line treatment. Safety and efficacy were confirmed by showing the oral drug significantly prolonged progression-free survival and overall survival times [16, 17]. The Food and Drug Adminstration in China (CFDA) has added apatinib to its list of approved drugs. This phase II, open-label, multicenter, single-arm trial was conducted to assess the safety and efficacy of apatinib as third-line or later treatment in patients with mGC.

## MATERIALS AND METHODS

### Patients

In all, 42 patients (10 male and 32 female; age 35-70 years [median 56]) with histologically confirmed mGC diagnosed at the cancer centers of four hospitals were enrolled from March, 2015 to October, 2015. They had at least one extracranial, not previously irradiated, measurable site of disease according to Response Evaluation Criteria in Solid Tumors (RECIST) 1.1 criteria; had received 1-4 regimens; failed second-line chemotherapy or the last chemotherapy regimen, and had no prior molecular targeted therapy. There were 11 and 21 patients with Eastern Cooperative Oncology Group (ECOG) scores of 1 and 2, respectively, 34 patients with 1 or 2 organ metastases, 8 patients with 2 or more organ metastases. The metastases were located in the liver in 29 patients, lung in 5 patients, and peritoneum in 8 patients.

Six of the 42 patients accepted radiotherapy. Twenty-eight and 14 patients received second-line chemotherapy and subsequent chemotherapy regimens, respectively. Clinical stage I, II, III, and IV mGC was diagnosed in 1, 1, 2, and 39 patients (Table 1).

**Table 1:** patient characteristics

Characteristics	N	%
Sex		
Male	32	76.2
Female	10	23.8
Median age years	56	
ECOG		
1	11	26.2
2	31	73.8
Prior surgery of primary tumor		
Yes	31	73.8
No	11	26.2
Stage		
II	1	2.4
III	2	4.8
IV	39	92.8
No of metastatic sites		
≤2	34	80.9
>2	8	19.1
Previous chemotherapy lines		
2	28	66.7
≥3	14	33.3
Metastasis site/organ		
Liver	29	69.0
Lung(s)	5	11.9
Posterior peritoneum lymph node	8	19.1
Prior radiotherapy		
Yes	6	14.3
No	36	85.7

### Study design

The aim of this open-label, single-arm, multicenter, phase II study was conducted to evaluate the safety and efficacy of apatinib as third-line or later treatment in patients with mGC and to provide reliable clinical trial data that would inform the design of a large multicenter phase III trial.

### Enrollment

#### Inclusion criteria

(1) age ≥18 years, (2) ECOG ≤2 , (3) second-line treatment failure, (4) expected survival time more than three months, (5) at least one measurable lesion according to RECIST 1.1 criteria, (6) acceptable liver and kidney function with treatment, and (7) provision of informed consent.

#### Exclusion criteria

(1) uncontrolled hypertension (140/90 mmHg or higher), (2) serious coronary heart disease (CHD), (3) treatment with anticoagulants and hemostatic drugs, (4) receiving radiotherapy, and (5) pregnancy or lactation.

### Method and dose

Based on the pharmacokinetic and bioavailability data obtained from the phase I clinical trial, apatinib 850 mg daily was administered 30 minutes postprandially on days 1 through 28 of each 4-week cycle. The apatinib was supplied by Jiangsu HengRui Medicine Co., Ltd. The dose was reduced to 750 mg per day if patients experienced 3/4 grade adverse events or became intolerant to therapy, further reduced to 500 mg per day if these adverse events persisted, or discontinued if these adverse events persisted even at a dose of 500 mg per day.

### Efficacy and adverse events

Evaluation included physical examination, laboratory tests (CBCs and blood chemistry), and imaging tests (MRI or CT scan of measurable lesions) at baseline and every 2 cycles (8 weeks) until disease progression. The objective response rate (ORR) was defined as the rate that patients achieved complete remission (CR) or partial response (PR) by RECIST 1.0 criteria. Disease control rate (DCR) was defined as the rate that patients achieved CR, PR, and stable disease (SD) after at least 8 weeks. Progression-free survival (PFS) was defined as the time to tumor progression or death from any cause. OS was defined as the time to death or the last follow-up.

Adverse events (AEs) were assessed and graded in accordance with the Common Terminology Criteria for AEs, version 4.0. The safety evaluation was continued for 28 days after the last dose of apatinib or until recovery to grade 1 or 0 from any acute toxicities associated with apatinib. Blood chemistry and counts were checked weekly, and liver and kidney functions were evaluated every 2 weeks.

### The end point

The primary end point of this study was PFS. The secondary end points included OS, DCR, ORR, and adverse events. The study was terminated under the following circumstances: (1) the efficacy and safety could not be evaluated according to the study protocol; (2) subjects withdrew their informed consent; (3) disease progression; (4) drug toxicity could not be tolerated; and (5) pregnancy.

### Follow-up

Follow-up was by structured record review and telephone interview. Patients were observed until death, loss to follow-up, or end of study. The last time of follow-up was June 30, 2016.

### Statistical analysis

To estimate sample size, a 7-month accrual period and 10-month follow-up period were assumed. The study was designed with two-sided, α = 0.05, 80% power to detect a null median PFS (*n* = 40). Assuming a 10% dropout rate, the final accrual number was 44.

Survival and safety were assessed in patients who received at least one cycle of apatinib. PFS and OS were estimated using Kaplan-Meier analysis. The SPSS 17.0 was used for all statistical analyses.

## RESULTS

### Recruitment and demographics

**Figure 1:**
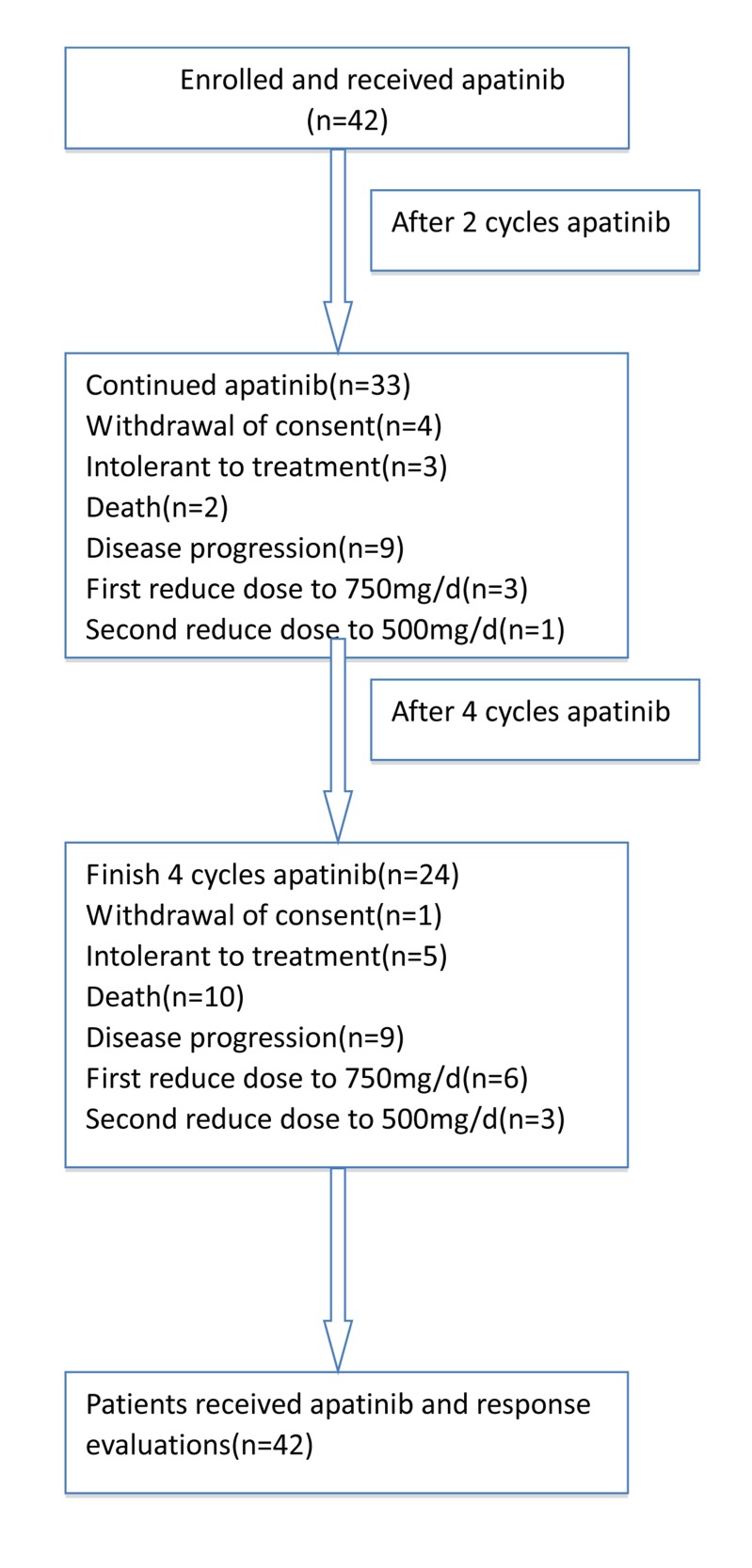
CONSORT Diagram, enrollment and outcomes

The study recruited 42 patients with mGC from 4 hospital cancer centers and was terminated on June 30, 2016. All patients received at least one cycle of oral treatment, and were evaluated after the second and fourth cycles, respectively. After the second cycle assessment, 4 patients withdrew, 3 patients experienced drug intolerance or adverse events, and 2 patients died. Drug dose was reduced to 750 mg per day in 3 patients and to 500 mg per day in one. A total of 33 patients received a third cycle. After four cycles, one patient dropped out of the study, 5 patients experienced drug intolerance or adverse events, and 10 patients died. Drug dose was reduced after the first cycle by 6 patients, the second cycle by 3 patients, and 24 patients completed all four cycles (Figure 1).

**Figure 2:**
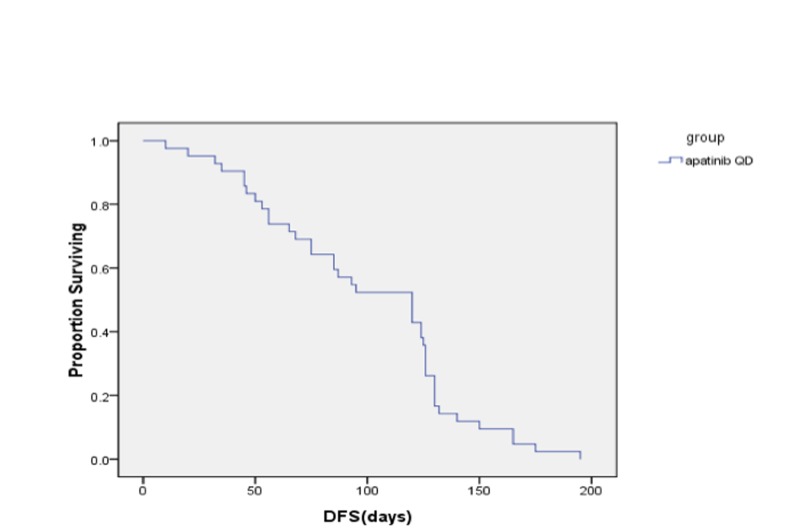
Progression survival table

**Figure 3:**
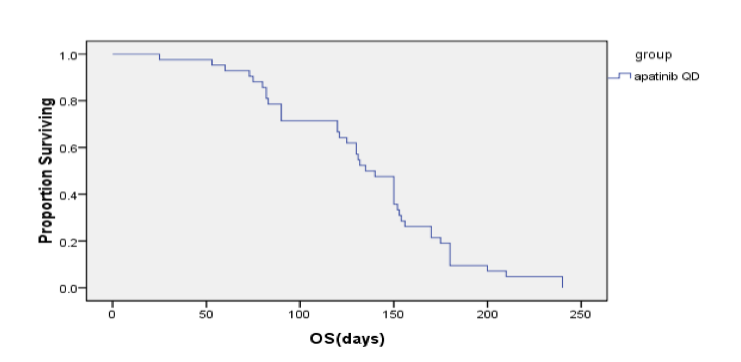
Overall survival

### Efficacy

At the date of last follow-up (June 30, 2016), all patients had undergone 1-4 cycles of chemotherapy, and achieved a median PFS time of 4.0 months (95% CI, 2.85-5.15) and median OS time of 4.50 months (95% CI, 4.03-4.97). After 2 cycles, there were 9, 29, and 9 patients who experienced PR, SD, and disease progression (PD), and the DCR and ORR were 78.57% and 9.52%, respectively. After 4 cycles, 1, 7, 16, and 9 patients had PR, SD, and PD, for a DCR of 57.14% and ORR of 19.05% (Table 2).

**Table2:** Disease response after treatment

	CR	PR	SD	PD	DCR %
After 2 cycles apatinib	0	4	29	9	33 (78.57%)
After 4 cycles apatinib	1	7	16	9	24 (57.14%)

### Safety

All patients were evaluated for drug toxicity and adverse events, which occurred in 38 patients to varying degree. These included secondary hypertension, hand-foot syndrome, fatigue, anemia, leukopenia, thrombocytopenia, diarrhea, vomiting, changes in the skin and mucous membranes, and so on. Toxicities were generally well tolerated. Secondary hypertension, elevated aminotransferase, and hand-foot syndrome were the main adverse reactions with incidence rates of 35.71%, 45.24%, and 40.48%, respectively. The secondary hypertension and elevated aminotransferase were the main grade 3/4 adverse events, each occurring in 3 patients and at an incidence rate of 7.14%. The total adverse event rate was 20.0% (Table 3).

**Table 3:** Adverse Event based on CTCAE 4.0

Adverse Event	Adverse Event Grade					
	0	1	2	3	4	Total (n,%)
Hypertension	27	8	4	3	0	15 (35.71)
Hand-foot syndrome	25	11	5	1	0	17 (40.48)
Fatigue	36	4	2	0	0	6 (14.29)
Diarrhea	35	4	2	1	0	7 (16.67)
Pain	35	3	3	1	0	7 (16.67)
Proteinuria	37	5	0	0	0	5 (11.90)
Nausea	37	2	1	1	0	5 (11.90)
Vomiting	30	8	4	0	0	12 (28.57)
Anemia	33	7	2	0	0	9 (21.43)
Leukopenia	27	7	8	0	0	15 (35.71)
Aminotransferase increased	23	9	7	3	0	19 (45.24)
Thrombocytopenia	32	6	3	1	0	10 (23.81)
Neutropenia	30	7	5	0	0	12 (28.57)
Dizziness	39	3	0	0	0	3 (7.14)

## DISCUSSION

Gastric cancer is among the most common malignancies in East Asian countries. Recurrence and metastasis are the biggest obstacles to successful treatment of gastric cancer. Eating habits are one of the important reasons. Medical conditions and diagnosis time are related to mortality. Many patients with gastric cancer already have metastases by the time they visit the hospital and so are no longer considered good candidates for surgery. The standard first-line treatment for mGC can slow disease progression and improve overall survival. New clinical evidence shows that some patients can benefit from secondary rescue chemotherapy [4-8, 22]. But for those who fail rescue treatment, there are a lack of effective treatments. Molecular targeted drugs bring new hope for those patients. Trastuzumab has been confirmed to be an effective treatment in patients with Her-2 gene positive breast cancer and gastric cancer [19, 20, 23]. However, dealing with Her-2 negative disease remains a clinical challenge. Apatinib is a small-molecule VEGFR tyrosine kinase inhibitor, similar to vatalanib (PTK787), but with a binding affinity 10 times that of vatalanib or sorafenib. It is a new oral molecular targeted drug that has been approved by the CFDA in China. This study investigated the efficacy of apatinib in patients with gastric cancer who experienced progression after receiving two or more lines of chemotherapy. It is an open-label, single-arm, multicenter, phase II study. All 42 patients enrolled in the study were treated with apatinib and evaluated. After taking one cycle of 850 mg per day for 28 days [24, 25], the median PFS and OS times were 4.0 months (95% CI, 2.85-5.15) and 4.50 months (95% CI, 4.03-4.97). Similarly, Li [25] reported that once daily 850 mg led to median PFS and OS times of 3.67 months and 4.83 months. In this study, the DCR and ORR were 78.57% and 9.52% after 2 cycles and 57.14% and 19.05% after 4 cycles. Adverse events (AEs) of varying degree occurred in 38 patients, including secondary hypertension, hand-foot syndrome, fatigue, anemia, leukopenia, thrombocytopenia, diarrhea, vomiting, changes in the skin and mucous membranes, and so on. The leading AEs (with incidence rates) were secondary hypertension (35.71%), elevated aminotransferase (45.24%), and hand-foot syndrome (40.48%). Grade 3 to 4 AEs were secondary hypertension (*n* = 3, 7.14%) and elevated aminotransferase (*n* = 3, 7.14%). The total AE rate was 20.0%. Drug dose was reduced in 9 patients because of grade 3 to 4 AEs, and treatment was terminated in 8 patients because of intolerance to AEs. Li [25] reported secondary hypertension and hand-foot syndrome as the main AEs and hematological toxicities of rarely more than grade 3. Hu [26] reported an incidence of grade 3 to 4 AEs (secondary hypertension and hand-foot syndrome) of 20.5% and 10.3%, respectively, in patients treated with apatinib for triple negative breast cancer. The incidence of AEs was higher in our study than in other studies, possibly because our mGC patients were in poorer condition and less tolerant.

In summary, this study was an open-label, single-arm, multicenter, phase II study of the safety and efficacy of apatinib (850 mg once daily). Patients given apatinib had high incidence of grade 3 to 4 AEs, which can be reduced by decreasing the dose.
